# A Suggestion to the Three Funds

**Published:** 1920-06-26

**Authors:** 


### The Hospital

The Workers' Newspaper of Administrative Medicine and Institutional Life, Administration, National Insurance and Health.

No. 1777, Vol. LXVIII. SATURDAY, JUNE 26, 1920. PRICE TWOPENCE

### A SUGGESTION TO THE THREE FUNDS.

It is sometimes the duty of a newspaper to set in print that which individual people only say in private, not in order to excite resentment, but in order to get behind the formal phrases which are necessarily the currency of intercourse. In the financial difficulties of the present year much has been heard of separate hospitals, much of some form of State support, but little of the three great Funds which stand between the London hospitals and the casual donors. To-day, however, the greatest of these Funds—King Edward's for London—has given a definite lead in the great emergency (p. 328).

Since the hospitals receive much from these Funds they are inevitably grateful, and could hardly, without apparent ingratitude, complain that they do not receive more. But inwardly there is dissatisfaction. As disinterested supporters of both, let us put on record that which does not find open words elsewhere. In a nutshell, the hospitals feel that each of them has often raised far more from one appeal than is raised by either Fund for their common benefit. Nor does the fact that each of the three Funds raises its quota yearly affect the argument, because the income of the larger hospitals individually is often more than that of either Fund. Let us take the figures. In 1919 the Hospital Sunday Fund raised £40,307, and received from legacies, investments, and special donations £45,924—a total of £86,231. The Hospital Saturday Fund, as our pages lately revealed, produced from all sources £78,000 last year. The possible income suggested by a correspondent in a reasoned argument—namely, £187,500—was not challenged as extravagant. The subscriptions to King Edward's Hospital Fund in 1919 were £25,199, and the receipts from all sources were £193,054. Add these totals together, and we find that the three Funds produced £357,285 in one year.

This is the best that London can do at present, and it would be idle to say that it is not felt to be poor and that complaints are not privately made about it.

Of the three great Funds, King Edward's Hospital Fund may, perhaps, legitimately be regarded as a reservoir to which contributions are expected to flow and which does not exist primarily to seek them out, though its functions of criticism and investigation do undoubtedly stimulate the confidence and the generosity of many wealthy philanthropists. The Hospital Sunday Fund and the Hospital Saturday Fund, on the other hand, are active organising and propagandising concerns, whose yield is a mathematical function of the energy and method employed to raise it. The endeavours and the difficulties of the Hospital Saturday Fund have been lately discussed in The Hospital. We need not here repeat the analysis already provided. With the difficulties and endeavours of the Hospital Sunday Fund we deal in the present number.

The general Funds, with their collective appeals, do not produce the results which special appeals bring to separate hospitals. If the result is regretted by the latter, there is an excuse which should be remembered in the proved weakness of a collective appeal. We admit this, but must argue that the efforts which are being made successfully by such hospitals as St. Bartholomew's and the London must be made by the great Funds also. If they can hardly have the personal initiative behind them that a separate appeal can have, the Funds do possess an organisation, and this must be developed and improved. For many think that the Funds are not pulling their weight at the moment. It is argued that the Sunday Fund, the Saturday Fund, in spite of recent progress, and the League of Mercy are standing where they stood before the war, and are making no effort comparable to that which individual hospitals are making to relieve the financial position in London. It may seem hard to include the Hospital Saturday Fund in this opinion because it has invited its sister bodies, as we note elsewhere, to combine in a united appeal during a "Hospital Week" to be held later in the year. But this special effort, useful though we hope it may prove if adopted, can be only half the task. The League of Mercy, the Sunday Fund, the Saturday Fund, if they make a concerted appeal in the autumn, must not neglect any means to improve their normal income. For even if the "Hospital Week" is held and a substantial sum is collected, unless "Hospital Week" becomes an annual event, the hospitals will be in the same position a year hence as they are now. We do not want to make too much of this argument, for voluntary finance is conducted from year to year, and does not pretend to look far ahead.

But each of the great Funds at the moment needs a policy just as much as the separate hospitals do. The trouble is that, being collecting and not spending bodies, they cannot feel the pinch as private hospitals do. If the income of a fund falls, so much the worse for other people. Thus the general hospitals of London look to the three Funds to make an effort comparable to that which individual hospitals are making. We think that view is reasonable, and urge an argument which no one else can urge without risk of misunderstanding.

